# Clinical, immunological and metabolomic risk factors associated with fibromyalgia in a cohort of patients with idiopathic inflammatory myopathies

**DOI:** 10.3389/fimmu.2026.1787182

**Published:** 2026-04-24

**Authors:** Beatriz Alcalá-Carmona, Samuel Govea-Peláez, Jiram Torres-Ruiz, Daniel Alberto Carrillo-Vázquez, Jennifer T. Balderas Miranda, Yatzil Reyna-Juárez, María José Ostos-Prado, Nancy R. Mejía-Domínguez, Guillermo Juárez-Vega, José Carlos Paez-Franco, Hilda Sánchez-Vidal, Karina Santana-De Anda, Ricardo Alejandre-Aguilar, José L. Maravillas-Montero, Diana Gómez-Martín

**Affiliations:** 1 Department of Immunology and Rheumatology, Instituto Nacional de Ciencias Médicas y Nutrición, Mexico, Mexico; 2 Red de Apoyo a la Investigación, Universidad Nacional Autónoma de México, Mexico, Mexico; 3 Laboratory of Entomology, Department of Parasitology, Escuela Nacional de Ciencias Biológicas, Instituto Politécnico Nacional, Mexico, Mexico; 4 Departamento de Medicina Molecular y Bioprocesos, Instituto de Biotecnología, Universidad Nacional Autónoma de México, Cuernavaca, Mexico

**Keywords:** adaptive immunity, chronic pain, cytokines, fibromyalgia, idiopathic inflammatory myopathies, metabolomics

## Abstract

**Objective:**

To investigate the clinical, immunological, and metabolomic factors associated with fibromyalgia (FM) in patients with idiopathic inflammatory myopathies (IIM) who are in clinical remission or complete response.

**Methods:**

In this cross-sectional, 49 patients with IIM meeting remission and/or complete clinical response criteria were evaluated with the PROMIS Pain Interference Short Form 8a as an initial screening tool and patients with clinically significant pain interference subsequently underwent assessment with the 2016 ACR criteria. Clinical data, flow cytometry of peripheral blood mononuclear cells, multiplex cytokine assays, and untargeted metabolomic profiling by GC-MS were performed. Multivariate logistic regression was used to identify variables associated with FM.

**Results:**

The prevalence of FM in this IIM cohort was 40.8%. FM was associated with higher patient global assessment scores, increased muscle damage, current prednisone use, and elevated serum levels of IL-6 and MCP-1. Immunophenotyping revealed reduced numbers of non-classical monocytes, CD8^+^ T cells, and B lymphocytes in FM patients. Metabolomic analysis identified lower concentrations of tryptophan and nonanoic acid in the FM group, suggesting altered pathways of immune regulation and nociplastic pain.

**Conclusion:**

Patients with IIM in remission and/or complete clinical response can present with clinical significant FM, which is associated with immune dysregulation and metabolic alterations. These findings highlight the need for routine FM screening in IIM and support the use of patient-reported outcomes to distinguish between inflammatory and nociplastic symptoms in clinical practice.

## Key messages:

- Patients with IIM and FM exhibit a distinct immune signature marked by reductions in non- classical monocytes, CD8^+^ T cells, and B cells, supporting a state of immune dysregulation that may underlie chronic pain amplification.
- Elevated serum levels of IL-6 and MCP-1 in these patients point to persistent low-grade inflammation driving central sensitization and nociplastic pain, even in clinical remission or complete clinical response.
- Lower concentrations of nonanoic acid, a metabolite involved in immunoregulation, suggest a potential link between host metabolism and pain susceptibility in autoimmune disease.

## 1 Introduction

Idiopathic inflammatory myopathies (IIM) are a group of systemic autoimmune disorders characterized by myositis, and a range of systemic manifestations affecting mainly the skin, lungs, and joints (1). These features often lead to functional decline and chronic pain as long-term sequelae (2). The International Myositis Assessment and Clinical Studies (IMACS) group has developed and validated the Core Set Measures (CSM) to assess disease activity in IIM (3). These measures include the Health Assessment Questionnaire (HAQ) and the Myositis Disease Activity Assessment Tool (MDAAT), which incorporate patient-reported symptoms such as fatigue and myalgias.

In 2019, the Outcome Measures in Rheumatology (OMERACT) Myositis Special Interest Group (SIG) aimed to identify the domains of greatest concern for patients and healthcare professionals. This international effort revealed that the most prioritized domains among patients (n=510), compared to healthcare providers (n=101) and caregivers (n=27), were fatigue (85.9%, p<0.0001), cognitive dysfunction (41.8%, p<0.0001), and insomnia (39.0%, p<0.0001) (4). However, a systematic review published in 2023 (5) found that none of the included studies directly evaluated the relationship between disease activity and pain. This highlights a critical gap in the comprehensive management of IIM—specifically, the need to assess the presence of comorbid conditions that may contribute to chronic pain, such as fibromyalgia. Patients with systemic autoimmune diseases (SAD) have a reduced health-related quality of life (HRQoL) compared to the general population, and chronic pain is a major contributing factor. In the MyoPAD study, which included qualitative interviews with 18 IIM patients, participants reported four dominant themes: fatigue, pain, daily symptom variability, and persistent symptoms despite normal muscle enzyme levels or preserved muscle strength upon examination (6). These findings reinforce the notion that chronic pain is a major unmet need in IIM management. However, the specific contribution of fibromyalgia—a condition characterized by widespread pain, fatigue, non-restorative sleep, and cognitive symptoms—has yet to be thoroughly investigated in this population.

Fibromyalgia (FM) is frequently associated with other rheumatologic conditions, with an estimated prevalence of 11–30% (7). Its coexistence with diseases such as rheumatoid arthritis (8), ankylosing spondylitis (9), and systemic lupus erythematosus (SLE) (10) is well-documented. Nonetheless, most studies have included few or no patients with IIM, limiting our understanding of FM in this specific context.

Multiple pathophysiological hypotheses have been proposed to explain chronic pain in FM, many of which involve dysregulation of the neuroimmune axis. Elevated serum concentrations of pro- inflammatory mediators such as CD40, IL-8, TNF, and CD5 have been observed in FM patients (11) Furthermore, proteomic analyses have identified increased levels of 19 inflammatory proteins, including IL-8, CCL3, TNF, IL-18R1, and EN-RAGE (12). To identify the cellular origin of these mediators, a murine model showed that expansion of non-classical monocytes (CD14^+^/CD16^++^) was positively correlated with resting pain, movement-evoked pain, and global fatigue (13). However, the role of other immune cell subsets in FM has not been comprehensively addressed, and most of this evidence excludes patients with FM in the setting of systemic autoimmune diseases such as IIM. Screening for fibromyalgia symptoms in IIM patients is essential, as the presence of FM significantly impacts quality of life and may warrant a multidisciplinary, non-immunosuppressive treatment approach. Moreover, FM-related symptoms may influence physician- and patient-reported activity and damage scores, potentially leading to overtreatment if misattributed to inflammatory disease activity. The identification of potential biomarkers of FM in the context of IIM could enable earlier detection and appropriate clinical management.

The aim of this study was to analyze the clinical, immunological, and metabolomic risk factors associated with fibromyalgia in patients with idiopathic inflammatory myopathies.

## 2 Methods

We conducted a cross-sectional study at a tertiary referral center in Mexico City, including patients with a previous diagnosis of idiopathic inflammatory myopathies (IIM) based on the 2017 EULAR/AC classification criteria (14) and the Connor criteria (15) for the diagnosis of anti-synthetase syndrome (AS) (16). All participants were part of the institutional Myositis Translational Research Cohort Salvador Zubirán (MYOTReCSZ). The study was approved by the Institutional Ethics and Research Committees (REF NUMBER 4749), in accordance with the Declaration of Helsinki, and all patients provided written free and informed consent prior to inclusion.

Patients were eligible if they met criteria for complete clinical response (≥6 months without evidence of disease activity and no changes in immunosuppressive therapy) or remission (≥6 months without disease activity and no immunosuppressive treatment) (17), as assessed during routine clinical follow- up by the certified rheumatologists (DGM, JTR). Clinical evaluation included the IMACS Core Set Measures (CSM): Patient Global Assessment (PtGA), Physician Global Assessment (PhGA), Manual Muscle Testing (MMT8), Health Assessment Questionnaire (HAQ), Myositis Disease Activity Assessment Tool (MDAAT), muscle enzyme levels, and the Myositis Damage Index (MDI). Additionally, all patients completed the PROMIS Pain Interference Short Form 8a (PISF) (18), which has been previously validated for use in IIM by the OMERACT Myositis Special Interest Group (SIG) (19). PROMIS Pain Interference was used as an initial screening instrument to identify patients reporting clinically significant pain interference during routine clinical follow-up. Patients with PROMIS Pain Interference scores >11 subsequently underwent evaluation using the 2016 ACR fibromyalgia classification criteria, including the Widespread Pain Index (WPI) and Symptom Severity Score (SSS) (18); this stepwise strategy was implemented to improve feasibility during clinical visits, however because the full 2016 ACR criteria were not systematically applied to all participants, under ascertainment of fibromyalgia in patients with lower PROMIS scores cannot be excluded. Controls were defined as IIM patients with a PISF score <11. We excluded individuals with active disease, current pregnancy, renal or hepatic impairment, overlap syndromes, and neoplastic disease; active infections were excluded through clinical evaluation by the treating rheumatologists, including symptom review, physical examination, and routine laboratory testing performed during the clinical visit. Demographic and clinical data were obtained from institutional medical records; current medications were recorded at the time of clinical evaluation, including glucocorticoids, conventional immunosuppressants (methotrexate, mycophenolate mofetil, and antimalarials), and biologic therapies. Prednisone use and current prednisone dose were included in the regression analyses. Other immunomodulatory therapies were explored descriptively and in exploratory analyses according to their frequency in the cohort. Peripheral blood samples. (20 mL) were obtained during routine morning outpatient visits under standardized clinical conditions. Although fasting status was not strictly required, samples were collected at similar times of day to minimize circadian variability in cytokine and metabolomic measurements; healthy donors were included for the metabolomic assessment, and were matched by sex and approximate age distribution ( 5 years) to each patient of the cohort.

### 2.1 Peripheral blood mononuclear cell immunophenotyping by flow cytometry

PBMCs were isolated by density gradient with centrifugation using Ficoll-Paque (GE Healthcare Life Sciences, Chicago, IL, USA). Following two washes with phosphate-buffered saline (PBS), cells were stained with Zombie Aqua viability dye (BioLegend, San Diego, CA, USA). After two additional washes with 5% fetal bovine serum (FBS) in PBS, cells were incubated for 30 minutes at room temperature with Fc receptor blocking reagent (Human TruStain FcX, BioLegend), followed by staining with the following fluorochrome-conjugated antibodies: CD3-APC/Fire, CD4-AF488, CD8-PE/Dazzle, CD19-APC, CD25-BV421, FoxP3-BV421, CD14-PerCP, CD16-AF700, CD33-BV711, CD11b-PE Dazzle, CD66b-PE/Cy7, HLA-DR-APC Fire, PD-L1-BV650, and Arginase-1-PE. All antibodies were purchased from BioLegend (San Diego, CA, USA). Data acquisition was performed on a 4-laser LSR Fortessa flow cytometer (BD Biosciences, Franklin Lakes, NJ, USA), with one million events recorded per sample. Analysis was conducted using FlowJo software (BD Biosciences). Absolute counts of low- density granulocytes (LDGs), myeloid derived suppressor cells, monocyte subsets, and lymphocyte populations were calculated based on complete blood count data obtained on the same day as PBMC isolation.

Immune cell subpopulations were defined as follows: CD4^+^ T cells (CD3^+^CD4^+^), CD8^+^ T cells (CD3^+^CD8^+^), CD4^+^ regulatory T cells (CD3^+^CD4^+^CD25^+^FoxP3^+^), CD8^+^ regulatory T cells (CD3^+^CD8^+^FoxP3^+^), B cells (CD3^-^CD19^+^), classical monocytes (CD14^++^CD16^-^), intermediate monocytes (CD14^++^CD16^+^), non-classical monocytes (CD14^+^CD16^++^), granulocytic myeloid-derived suppressor cells (G-MDSCs: CD33^+^CD11b^+^CD66b^+^CD14^-^HLA-DR^low^/^-^), monocytic MDSCs (M MDSCs: CD33^+^CD11b^+^CD14^+^CD66b^-^HLA-DR^low^/^-^), and low-density granulocytes (LDGs: CD66b^+^CD16^+^CD14^-^HLA-DR^-^).

### 2.2 Normal density granulocytes immunophenotyping by flow cytometry

After blood separation by density gradients, normal density granulocytes (NDGs) were isolated with dextran sedimentation. Red blood cells were lysed with a hypotonic solution, and after two washes with phosphate-buffered saline (PBS), cells were stained with Zombie Aqua viability dye (BioLegend, San Diego, CA, USA). After two additional washes with 5% fetal bovine serum (FBS) in PBS, cells were incubated for 30 minutes at room temperature with Fc receptor blocking reagent (Human TruStain FcX, BioLegend), followed by surface staining with the following fluorochrome-conjugated antibodies: CD15-PE, CD62L-PerCP/Cy5.5, CD11a-FITC, CD11b-PE/Dazzle, CD11c-BV711, CD177-APC, and CD16-AF700. Intracellular staining for IL-10 was performed after fixation and permeabilization using the Cytofix/Cytoperm kit (BD Biosciences), followed by incubation with IL-10-BV421 (BioLegend).

Granulocyte subpopulations were defined as follows: Naïve neutrophils CD15^+^CD62L^+^CD11b^low^CD16^-^), mature neutrophils: (CD15^+^CD62L^+^CD11b^+^CD16^+^), regulator neutrophils (CD15^+^CD11b^+^CD177^+^IL-10^+^) and transmigrated neutrophils (CD15^+^CD62L^low^CD11a^+^CD11c^+^).

### 2.3 Pro-inflammatory cytokine and chemokine assessment

Serum concentrations of cytokines and chemokines were quantified using the Bio-Plex Pro™ Human Cytokine Group I Panel 17-plex assay (Bio-Rad Laboratories, Hercules, CA, USA; catalog number M5000031YV) according to the manufacturer’s instructions. This magnetic bead–based multiplex immunoassay simultaneously measures 17 analytes (IL-1β, IL-2, IL-4, IL-5, IL-6, IL-7, IL-8, IL-10, IL-12p70, IL-13, IL-17A, G-CSF, GM-CSF, IFN-γ, MCP-1, MIP-1β, and TNF-α) using the Bio-Plex/Luminex platform. Calibration curves were generated using premixed standards supplied by the manufacturer, and assay quality control procedures were performed according to the manufacturer’s recommendations. Cytokine concentrations were reported in pg/mL, as provided by the Bio-Plex analysis software.

### 2.4 Metabolomic assessment

Serum metabolomic profiling was performed by gas chromatography–mass spectrometry (GC-MS) (37). Briefly, 35 μL of serum were mixed with 150 μL of a chloroform-methanol solution (1:3, v/v) and 5 μL of an internal standard mix (tridecanoic acid, methyl tricosanoate, and 5α-cholestane at 0.18 mg/mL each). The mixture was vortexed for 2 minutes, incubated at –20 °C for 20 minutes, and centrifuged at 16,000 × g for 10 minutes at 4 °C. The supernatant was collected, evaporated under nitrogen, and derivatized first with 40 μL of methoxyamine in pyridine (90 min at 37 °C), followed by 40 μL of MSTFA + 1% TMCS (30 min at 37 °C). One microliter of the final solution was injected in splitless mode into a GC-MS system (Agilent 5977A/7890B) equipped with an autosampler (G4513A) and an HP-5ms capillary column (30 m × 250 μm × 0.25 μm). Helium (99.9999%) served as the carrier gas at a constant flow of 1 mL/min. The oven temperature program was: initial hold at 60 °C for 1 min, ramp to 325 °C at 10 °C/min, final hold for 10 min. Sample injections were randomized. A pooled quality control (QC) sample was analyzed every six injections to monitor instrument performance and reproducibility. Metabolites with a relative standard deviation (RSD) >30% in QC samples were excluded. A total of 45 metabolites met quality criteria. All solvents and reagents were of analytical grade.

### 2.5 Statistical analysis

Sample size calculation was performed based on previously reported differences in serum IL-10 concentrations between patients with fibromyalgia (mean 24.5 ± 33.06) and healthy controls (mean 1.06 ± 3.54).(1) Using these parameters, an effect size of 0.79 was estimated. Assuming a two sided α of 0.05 and a statistical power of 80%, the required sample size was calculated as 30 subjects per group, including a 10% allowance for potential losses. Because no prior data were available for the main immunological and metabolomic variables evaluated in IIM patients with fibromyalgia, this calculation was used as an approximate reference for a two-group biomarker comparison. However, it does not specifically power the multivariable logistic regression analyses or the simultaneous evaluation of multiple immunological and metabolomic variables included in the present study. Continuous variables were expressed as medians with interquartile ranges (IQR). Categorical variables were compared using the chi-square test. The Mann–Whitney U test was used to compare continuous variables between groups. Variables with significant association (p < 0.05) in univariate logistic regression were selected for multivariate analysis. Stepwise selection based on the Akaike Information Criterion (AIC) was used to identify variables associated to fibromyalgia in multivariate logistic regression. Results were reported as odds ratios (OR) with 95% confidence intervals and corresponding p-values. Statistical analyses and sample size calculations were performed using R software (R Core Team, 2021. R Foundation for Statistical Computing, Vienna, Austria. http://www.R-project.org/). Principal Component Analysis (PCA) and Partial Least Squares– Discriminant Analysis (PLS-DA) were conducted using MetaboAnalyst 5.0 (20), and after sum normalization, log transformation, and mean centering. Data normalization using the internal standard (tridecanoic acid) demonstrated consistency, confirming the robustness of the preprocessing workflow.

## 3 Results

A total of 49 patients with a diagnosis of idiopathic inflammatory myopathy (IIM) in complete clinical response or remission were included between November 2023 and April 2024 (**Table 1**). Because the present study specifically targeted patients in remission or complete clinical response, the number of eligible participants during the recruitment period was limited. Among them, 39 were women (79.5%) with a median age of 51 years. The distribution of IIM subtypes included 39 patients (79.5%) with dermatomyositis, 7 (14.2%) with antisynthetase syndrome, and 3 (6.1%) with polymyositis. The prevalence of fibromyalgia (FM), based on the 2016 ACR/EULAR criteria, was 40.8% (20/49 patients). Patients with FM were more frequently female (95% vs. 68.9% in the non-FM group, p = 0.016). No significant differences were found regarding age or body mass index (BMI). Mi-2 was the most frequent antibody in both groups (30% in IIM with FM vs. 27.5% in IIM without FM).

**Table 1:** Demographic, clinical and immunological features in IIM patients with or without a diagnosis of FM.

Variable	IIM patients with FM (n=20)	IIM patients without FM (n=29)	P value
Demographic factors (median, IQR)			
Age (y)	52 (44-59.50)	51 (33-58)	NS
Female sex (%)	19/20 (95%)	20/29 (68.90%)	0.016*
BMI (kg/m^2^)	27.00 (23.10-30.30)	26.20 (21-29.60)	NS
Dermatomyositis (%)	15/20 (75%)	22/29 (75.80%)	NS
Antisynthetase syndrome (%)	4/20 (20%)	3/29 (10.30%)	NS
Other (%)	1/20 (5%)	2/29 (6.80%)	NS
Months from diagnosis to inclusion (m)	59.50 (45-122)	60 (28-89)	NS
Clinical factors (median, IQR)			
Interstitial lung disease (%)	6/20 (30%)	3/29 (10.30%)	0.08
Calcinosis (%)	0/20 (0%)	5/29 (17.20%)	0.017*
PROMIS SF 8a (8-40)	16.50 (12.75-20.25)	8 (8-9)	<0.001 *
WPI (0-19)	7.50 (4-10)	NA	NA
SSS - total (0-12)	6.50 (4.75-8)	NA	NA
SSS - fatigue (0-3)	2 (1-2)	NA	NA
SSS – sleep (0-3)	2 (1-2)	NA	NA
SSS – cognitive symptoms (0-3)	1 (0-2)	NA	NA
MMT8–80 score (0-80)	80 (79-80)	80 (80-80)	NS
Patient global assessment VAS (0-10)	3.50 (2-5.25)	0.50 (0-2)	0.001*
Physician global assessment VAS (0-10)	0 (0-1.25)	0 (0-0)	NS
Muscular damage global assessment VAS (0-10)	1.50 (1-3.25)	1 (0-1)	0.023*
Muscular damage score (%)	1.50 (0-3)	0 (0-1)	0.036*
Treatment (median, IQR)			
Methotrexate (n, %)	11/20 (55%)	13/29 (44.80%)	NS
Methotrexate – dose (mg)	12.50 (0-25)	0 (0-15)	NS
Mycophenolate mofetil (n, %)	7/20 (35%)	7/29 (24.10%)	NS
Mycophenolate mofetil – dose (mg)	0 (0-1500)	0 (0-500)	NS
Antimalarial (n, %)	12/20 (60%)	17/29 (58.60%)	NS
Antimalarial – dose(mg)	200 (0-300)	150 (0-200)	NS
Prednisone (n, %)	11/20 (55%)	5/29 (17.20%)	0.005*
Prednisone – dose (mg)	3.75 (0-5)	0 (0-0)	0.004*
Prednisone – cumulative dose (mg)	11.40 (8.05-20.12)	12.20 (3.20-15.80)	NS

*significant p value <0.05.

### 3.1 Clinical features associated with FM in IIM patients

No differences among IIM subtype, or disease duration were found according to the FM diagnosis. Notably, none of the patients with FM exhibited calcinosis, compared to 17.2% (5/29) of patients in the non-FM group (p = 0.017). Regarding daily functioning, FM patients reported significantly greater pain interference, with higher scores on the PROMIS 8a Pain Interference Questionnaire (median 16.5 [12.75–20.25] vs. 8 (8, 9), p < 0.001). Within the Widespread Pain Index (WPI) and Symptom Severity Scale (SSS), the most affected symptom in FM patients was chronic fatigue (median 2 [1.0–2.0]). Patients with FM exhibited higher disease perception and tissue damage scores. The patient global assessment (PGA) was significantly higher in the FM group (3.5 vs. 0.50, p = 0.001), as were the global assessment of muscle damage (1.5 vs. 1.0, p = 0.023) and the Muscle Damage Index (1.5 vs. 0, p = 0.036). Regarding pharmacological treatment, biologic therapies in this cohort were limited to B-cell depletion therapy one year prior to inclusion (n=3), and no patients were receiving JAK inhibitors or other cytokine-targeted biologics at the time of inclusion. FM patients had greater exposure to glucocorticoids. A higher proportion were receiving prednisone (55% vs. 17.2%, p = 0.005), with a higher median current dose (3.75 mg vs. 0 mg, p = 0.004).

### 3.2 Patients with IIM and FM displayed a differential immunological profile

Serum IL-6 levels (**Table 2**) were elevated (0.11 vs. 0.03 pg/mL, p = 0.03), and there was a reduction in several PBMCs subsets **Table 3**), specifically, non-classical monocytes (16.4 vs. 28.5 cells/μL, p = 0.027), CD8^+^ T cells (174 vs. 243 cells/μL, p = 0.045), percentage of B cells (5% vs. 10.5%, p = 0.015), and absolute B cell counts (58.4 vs. 152 cells/μL, p = 0.005).

**Table 2:** Pro-inflammatory Cytokines and Chemokines in IIM patients with or without a diagnosis of FM.

Variable	IIM patients with FM (n=20)	IIM patients without FM (n=29)	P value
Cytokine/Chemokine (median, IQR)			
IL-1β (pg/mL)	0 (0.00-0.03)	0.03 (0.00-0.03)	NS
IL-2 (pg/mL)	0.00 (0.00-0.60)	0.00 (0.00-0.00)	NS
IL-4 (pg/mL)	0 (0.00-0.00)	0 (0.00-0.00)	NA
IL-5 (pg/mL)	0 (0.00-0.00)	0 (0.00-0.00)	NA
IL-6 (pg/mL)	0 (0.00-0.02)	0 (0.00-0.00)	0.023*
IL-7 (pg/mL)	2.68 (0.0.44-4.43)	1.45 (0.44-3.78)	NS
IL-8 (pg/mL)	1.37 (0.62-2.98)	1.20 (0.49-2.04)	NS
IL-10 (pg/mL)	0.12 (0.00-0.27)	0.12 (0.00-0.27)	NS
IL-12p70 (pg/mL)	0 (0.00-0.00)	0 (0.00-0.00)	NS
IL-13 (pg/mL)	0 (0.00-0.00)	0 (0.00-0.00)	NA
IL-17 (pg/mL)	0.33 (0.18-0.38)	0.22 (0.08-0.37)	NS
G-CSF (pg/mL)	0.89 (0.00-2.51)	0.59 (0.00-3.5)	NS
GM-CSF (pg/mL)	0 (0.00-0.00)	0 (0.00-0.00)	NA
IFN-ɣ (pg/mL)	0.20 (0.17-0.30)	0.20 (0.20-0.30)	NS
MCP-1 (pg/mL)	4.6 (2.40-14.32)	3.31 (1.49-6.37)	NS
MIP-1b (pg/mL)	11.73 (5.14-15.3)	10.1 (4.87-19.93)	NS
TNF-ɑ (pg/mL)	0 (0.00-0.02)	0 (0.00-0.00)	NS

*significant p value <0.05.

**Table 3:** Cellular subsets quantification in IIM patients with or without a diagnosis of FM.

Variable	IIM + FM patients (n=20)	IIM without FM patients (n=29) (n=29)	P value
Neutrophils (median, IQR)			
Regulatory neutrophils (%)	0.11 (0-1.31)	0.07 (0-0.290)	NS
Regulatory neutrophils (cells/µl)	2.58 (0.09-56.80)	1.78 (0.21-9.68)	NS
Naive neutrophils (%)	54.40 (37.42-61.70)	51.10 (30.50-68.90)	NS
Naive neutrophils (cells/µl)	1813.10 (1151.40- 2677.80)	1599.40 (747.80- 2694)	NS
Low density granulocytes (median, IQR)			
Low density granulocytes (%)	4.50 (2.27-7.20)	7 (2.43-10.77)	NS
Monocytes (median, IQR)			
Intermediate monocytes (%)	5.10 (3.93-6.38)	4.60 (2.79-7.72)	NS
Intermediate monocytes (cells/µl)	25.10 (12.32-36.80)	19.80 (15.80-41.20)	NS
Non classical monocytes (%)	3.60 (1.94-5.51)	6.10 (3.53-9.06)	0.053
Non classical monocytes (cells/µl)	16.40 (8.30-24.30)	28.50 (13.70-53.70)	0.027*
Lymphocytes (median, IQR)			
T CD8+ lymphocytes (%)	15.15 (7.50-26.23)	18.60 (12.78-27.05)	NS
T CD8+ lymphocytes (cells/µl)	174.04 (94.23-292.78)	243 (183-479.85)	0.045*
Treg CD8+ lymphocytes (%)	0.02 (0-0.12)	0.05 (0.01-0.13)	NS
Treg CD8+ lymphocytes (cells/µl)	0.04 (0.01-0.38)	0.13 (0.05-0.42)	NS
B lymphocytes (%)	5.04 (1.72-9.85)	10.50 (6.93-13.90)	0.015*
B lymphocytes (cells/µl)	58.44 (21.13-143.35)	152 (82.14-272.34)	0.005*

*significant p value <0.05.

### 3.3 Metabolomic signature in patients with IIM and FM

**Figure 1:**
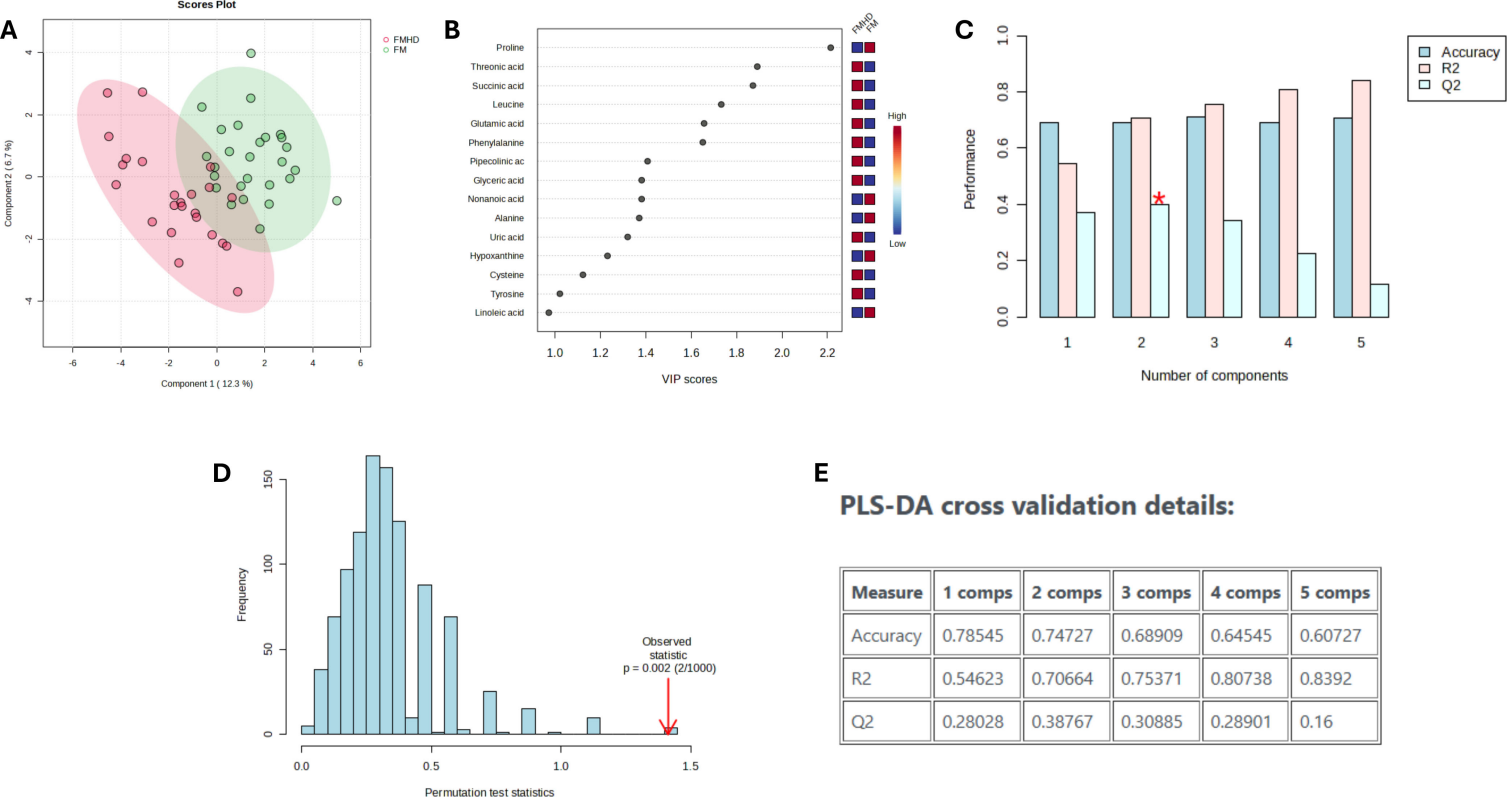
PLS-DA analysis comparing HD, healthy donors and patients with idiopathic inflammatorymyopathies without fibromyalgia (IIM). **(A)** PLS-DA scores plot demonstrates clear group separation between healthy donors (pink) and IIM patients without FM (green) along Component 1 (12.3%) and Component 2 (6.7%). **(B)** VIP scores plot showing key discriminatory metabolites such as proline, threonic acid, succinic acid, leucine, and glutamic acid. **(C)** Cross-validation metrics indicate optimal model performance with two components (Accuracy = 0.75, Q² = 0.39). **(D)** Permutation test confirms statistically significant separation between groups (p = 0.002). **(E)** PLS-DA model performance summary table.

The healthy donor cohort consisted of individuals with a mean age of 44.8 years (median 47, range 25–69), showing a distribution centered in middle adulthood with representation across a broad adult age range. Partial least squares discriminant analysis (PLS-DA) was performed to evaluate metabolic differences between groups as described in Methods. The comparison between IIM patients without FM and healthy donors (HD) showed clear separation along component 1 (12.3%) and component 2 (6.7%), with good model metrics (accuracy = 0.75, R² = 0.71, Q² = 0.39) and a significant permutation test (p = 0.002). Key discriminatory metabolites included proline, succinic acid, leucine, glutamic acid, and threonic acid, indicating disturbances in amino acid and energy metabolism (**Figure 1**). The comparison between HD and IIM patients with FM showed moderate separation (component 1 = 10.8%, component 2 = 6.4%), with lower model performance (accuracy = 0.69, R² = 0.69, Q² = 0.18) and marginal permutation significance (p = 0.081). Overlap in discriminatory metabolites was observed, suggesting that the presence of FM contributes to greater metabolic variability. (**Supplementary Figure 1**). The direct comparison between IIM with and without FM revealed poor separation and low discriminative power (component 1 = 10.4%, component 2 = 8.3%; accuracy = 0.57, R² = 0.43, Q² = −0.23), with a non-significant permutation test (p = 0.331) (**Supplementary Figure 2**), suggesting that FM introduces heterogeneous metabolic shifts rather than a consistent discriminative profile. **Table 4**.

**Table 4:** Variables related to the association of FM in IIM patients.

Variable	Univariate analysis		Multivariate analysis	
	OR (CI 95%)	P value	OR (CI 95%)	P value
Patient global disease assessment (0-10)	1.84 (1.33-2.84)	<0.0001*	14.43 (2.44-360.79)	0.003*
Muscular damage global assessment (0- 10)	1.70 (1.33-2.84)	0.016*	7.80 (1.77-143.86)	0.02*
Lung damage score (0- 6)	3.71 (0.95-18.21)	0.058	2.94x10^-6^ (8.40x10^-15^-0.19)	0.28
MCP-1 (pg/mL)	1.07 (1.00-1.17)	0.042*	2.17 (1.07-8.17)	0.15
IL-8 (pg/mL)	1.29 (0.98-1.97)	0.064	0.30 (0.03-1.35)	0.59
Nonanoic acid	0.44 (0.15-1.12)	0.089	0.01 (1.47x10^-5^-0.40)	0.005*
Male sex (%)	0.11 (0.006-0.71)	0.016*		
Calcinosis (%)	2.82x10^-8^ (0-1.10x10^72^)	0.017*		
Muscle damage score (0-5)	1.63 (1.09-2.87)	0.02*		
PDN (%)	5.86 (1.66-23.41)	0.005*		
PDN dose (mg)	1.27 (1.05-1.64)	0.003*		
CNI dose(mg)	1.74 (1.07-6.79)	0.015*		
Percentage of B lymphocytes (%)	0.87 (0.77-0.97)	0.015*		
Absolute B lymphocytes(cells/µL)	0.99 (0.98-0.99)	0.006*		
IL-1β (pg/mL)	2.8x10^-13^ (4.51 x10^-30^-0.36)	0.022*		
IL-6 (pg/mL)	3.9x10^6^ (4.01-2.76 x10^21^)	0.009*		
Tryptophan	0.92 (0.84-0.99)	0.035*		

*significant p value <0.05.

## 4 Exploratory multivariable analysis of clinical, inflammatory, and metabolomic variables associated with FM in IIM patients

Univariate logistic regression analysis identified the following variables as positively associated with FM in IIM patients: PGA (OR 1.84, 95% CI 1.33–2.84, p < 0.0001), the visual analog scale (VAS) of muscle damage (OR 1.70, 95% CI 1.09–2.87, p = 0.016), muscle damage score (OR 1.63, 95% CI 1.07–2.65, p = 0.02), prednisone use (OR 5.86, 95% CI 1.66–23.41, p = 0.005), current prednisone dose (OR 1.27, 95% CI 1.05–1.64, p = 0.003), IL-6 levels (OR 3.96, 95% CI 4.0–27.6, p = 0.009), and MCP-1 levels (OR 1.07, 95% CI 1.00–1.75, p = 0.042). Variables negatively associated with FM included male sex (OR 0.11, 95% CI 0.006–0.71, p = 0.016) and absolute B cell count (OR 0.99, 95% CI 0.98–0.99, p = 0.006). In the exploratory multivariate logistic regression model, PGA (OR 14.4, 95% CI 2.44–360.79, p = 0.003), muscle damage GA (OR 7.80, 95% CI 1.77–143.86, p = 0.02), and MCP-1 levels (OR 2.17, 95% CI 1.07–8.17, p = 0.015) remained associated with FM. In contrast, nonanoic acid levels were identified as a protective factor (OR 0.01, 95% CI 1.47×10^-5^–0.40, p = 0.005). Prednisone use and current prednisone dose were associated with FM in univariate analyses but were not retained in the final multivariable model.

## 5 Discussion

Our findings demonstrate that a substantial proportion of patients with idiopathic inflammatory myopathies (IIM), despite being in remission or complete clinical response, continue to experience significant pain interference, with a FM prevalence of 40.8%. This represents one of the highest prevalences reported in autoimmune diseases and, to our knowledge, the largest cohort assessing FM prevalence in IIM using validated criteria. Notably, our methodology incorporated both the PROMIS Pain Interference scale, previously validated in IIM populations (19), as a pragmatic screening tool and the 2016 ACR revised criteria for FM diagnosis (21) in patients with clinically significant pain interference. While this approach was feasible in routine clinical practice, it may have led to under-ascertainment of fibromyalgia in some patients who were not formally evaluated using the full classification criteria.

This prevalence is notably higher than previously reported in systemic autoimmune diseases such as rheumatoid arthritis (11–20%) (8), ankylosing spondylitis (13–21%) (9), and systemic lupus erythematosus (17–25%) (10). While fibromyalgia has been studied in other rheumatic conditions, it remains understudied in the context of IIM; a recent prospective study assessing MSA and MAA positivity in FM patients without prior autoimmune disease found that up to 9% were seropositive, and 3% were later diagnosed with inflammatory myopathy (22). These findings suggest that FM may be both underrecognized and clinically relevant in the spectrum of IIM. However, in our cohort, we did not find any association between fibromyalgia and myositis-specific or myositis-associated autoantibodies, underscoring the need for further research to clarify the immunological links, if any, between these conditions.

From a clinical perspective, our results indicate that higher patient global assessment (PtGA), increased muscle, and higher corticosteroid use were associated with FM diagnosis in univariate analyses. The elevation in PtGA likely reflects the impact of pain, fatigue, and non-restorative sleep, all key components of FM, rather than ongoing inflammation. Previous studies in patients with SLE and RA have demonstrated that the presence of FM increases PtGA and physician global scores, potentially leading to overtreatment (8, 10). This phenomenon is particularly concerning in IIM, where misattributing FM symptoms to disease activity could perpetuate unnecessary immunosuppression.

Muscle and lung damage, although traditionally interpreted as irreversible structural complications, may exacerbate symptom burden and reinforce central sensitization pathways in FM. The chronicity and unpredictability of IIM symptoms—combined with functional disability and emotional distress— may contribute to the development or persistence of nociplastic pain syndromes. Similar associations have been reported in systemic sclerosis and SLE, where organ damage has been linked to heightened pain perception and functional limitation (5, 6).

The association between prednisone use and FM observed in our cohort mirrors findings in other autoimmune diseases. The association between prednisone use and FM observed in our cohort mirrors findings in other autoimmune diseases. However, prednisone use and current prednisone dose were associated with FM only in univariate analyses and were not retained in the final multivariable model. Therefore, glucocorticoid exposure should be interpreted primarily as a potential source of residual confounding rather than as a confirmed independent predictor. Because corticosteroids may influence immune cell distribution, cytokine signaling, sleep quality, fatigue, and symptom perception, their contribution to the observed immunological and clinical associations cannot be fully excluded. Chakr et al. reported that patients with RA and concomitant FM received higher glucocorticoid doses (5.0 vs. 2.5 mg/day, p<0.001), likely due to persistent symptoms being misattributed to active inflammation (23). This raises concerns about overtreatment and highlights the need for careful differentiation between inflammatory and nociplastic pain, as well as a possible influence for immune cell distribution and cytokine signaling (2). Although prednisolone is not known to directly cause FM, chronic corticosteroid use may contribute to sleep disturbances, fatigue, and mood changes, all of which can exacerbate FM symptomatology. In line with prior observations in RA where patients with FM were prescribed glucocorticoids more frequently and at higher doses (65.4% vs. 52.9%, p<0.001) (23) we also noted a similar prescription trend in IIM, although without significant differences in the use of other immunomodulators. These findings emphasize the importance of incorporating patient-reported outcomes, such as PROMIS, alongside conventional measures like MMT8 and CK, to better capture the patient’s lived experience, avoid misclassification of disease activity, and prevent unnecessary escalation of immunosuppressive therapy (19).

Regarding the immunological findings, patients with IIM and FM displayed a distinct immunophenotypic profile, including a significant reduction in non-classical monocytes (CD14^low^ CD16^+^), CD8^+^ T lymphocytes, and B cells. Non-classical monocytes are recognized for their patrolling behavior along the endothelium, their role in tissue surveillance, and anti-inflammatory regulation, including clearance of damaged cells and modulation of the endothelial integrity (24). In chronic inflammatory or autoimmune settings, sustained monocyte activation may lead to peripheral depletion of this subset due to migration toward inflamed tissues and altered myeloid differentiation (25). This has been observed in diseases such as systemic lupus erythematosus and rheumatoid arthritis, where reduced levels of circulating non-classical monocytes are attributed to chemokine-driven trafficking and immune exhaustion (26). Supporting our findings, a diminished pool of patrolling monocytes has been reported in FM patients, potentially related to immunosenescence and impaired regulatory mechanisms, both of which may contribute to persistent nociception and fatigue (27). Therefore, the observed decrease in non-classical monocytes in IIM patients with FM could reflect a failure in tissue- resolving monocyte responses, leading to an unbalanced pro-inflammatory environment and promoting chronic pain and poor resolution of inflammation in these patients.

The CD8+ T cell population was also significantly lower in IIM patients with FM. This finding is in line with previous reports that chronic widespread pain and FM are associated with a reduction in cytotoxic CD8^+^ T lymphocytes (28). Kaufmann et al, observed a significant drop in CD8^+^ T cells in patients with chronic pain, and noted an inverse correlation between posttraumatic stress symptom severity and CD8^+^ T cell numbers in FM patients. Such evidence supports the idea that chronic stress and pain, both prominent in FM, can lead to a suppressed cytotoxic T-cell compartment (28). It’s conceivable that this relative deficiency in CD8^+^ T cells, which can modulate immune responses and neuropathic pain, plays a role in the heightened pain sensitivity or fatigue seen in FM.

On the other hand, IL−6 levels were significantly higher in IIM patients with FM compared to those without. This aligns with meta-analytic data showing fibromyalgia patients have elevated plasma IL−6 levels compared to controls (11). Elevated IL−6 in FM correlates with pain intensity (29), and exogenous IL−6 administration in animal models induces hyperalgesia and FM-like symptoms (29). Mechanistically, IL−6 activates the JAK/STAT3 signaling pathway, leading to downstream chemokine production and neuroinflammation via post-translational STAT3 phosphorylation, thus potentially establishing a feed forward pro-algesic loop (30). Therefore, the elevated IL−6 observed in our FM- overlap cohort likely reflects an inflammatory milieu contributing to amplified pain perception, supporting the concept that FM, primary or secondary, bears a significant neuro-immune component in which IL−6 plays a critical role.

In contrast, Monocyte Chemoattractant Protein−1 (MCP−1/CCL2) emerged as a factor in our univariate regression (OR = 1.07, p = 0.042), suggesting an association with FM. Consistent with prior research, MCP−1 is significantly elevated in FM and chronic fatigue syndrome compared to controls, even when other cytokines are lower (31). As a potent monocyte/macrophage chemoattractant, MCP−1 fosters neuroinflammation via glial activation and has been linked to nociceptive sensitization; in FM, MCP−1 correlates with pain intensity (32). Although the increase in our cohort was modest, it mirrors patterns seen in primary FM and suggests that FM in IIM shares similar chemokine-driven inflammatory pathways. Together, IL−6 and MCP−1 define a pro-inflammatory signature in IIM patients with FM, systemic IL−6 elevation and MCP−1–mediated monocyte recruitment and neuroinflammation, highlighting the centrality of neuroimmune circuits in fibromyalgia pathophysiology.

The direct metabolomic comparison between IIM patients with and without fibromyalgia demonstrated poor group separation and low predictive performance, indicating that the metabolomic differences identified in this study should be interpreted cautiously and considered exploratory. Nevertheless, regression analyses identified an association between serum nonanoic acid levels and fibromyalgia status in patients with IIM. Nonanoic acid (pelargonic acid), a nine-carbon medium-chain fatty acid, has been reported to modulate immune responses through epigenetic mechanisms. Experimental studies suggest that this metabolite can upregulate host defense peptide expression by inhibiting histone deacetylases (HDACs), thereby increasing histone acetylation marks such as H3K9ac at β-defensin gene promoters and enhancing immune regulatory pathways (33). Such epigenetic effects may promote a regulatory, anti-inflammatory phenotype in innate immune cells. Additionally, nonanoic acid and related medium-chain fatty acids have been shown to suppress NF-κB–mediated inflammatory signaling and reduce pro-inflammatory cytokine production *in vitro* and in hepatic cell models (34). These compounds have also been reported to attenuate oxidative stress and MAPK pathway activation—mechanisms implicated in central sensitization and chronic pain—thereby reducing hyperalgesia in experimental settings (35). Consistent with this concept, targeted metabolomic analyses in patients with chronic widespread pain have identified increased circulating levels of nonanoic acid that inversely correlate with pain severity (36). However, although nonanoic acid levels were associated with fibromyalgia status in the regression analysis, this observation should be considered hypothesis- generating rather than evidence of a validated biomarker or causal mechanism. Further studies in larger cohorts and mechanistic models will be required to determine whether nonanoic acid plays a functional role in immune regulation or nociceptive pathways in IIM-associated fibromyalgia.

This study has several limitations that should be acknowledged. First, its cross-sectional design precludes the establishment of causal relationships between immune alterations, metabolomic changes, and fibromyalgia, and the observed associations may represent either contributing mechanisms or consequences of chronic nociplastic pain. Additionally, the cohort was derived from a single tertiary referral center and was predominantly composed of patients with dermatomyositis. Although this reflects the case-mix of our institutional myositis cohort, it may limit the generalizability of the findings to other populations or to less represented idiopathic inflammatory myopathy (IIM) subtypes. Also, fibromyalgia was formally assessed only in patients with PROMIS Pain Interference scores >11; therefore, under-ascertainment of fibromyalgia in some participants cannot be excluded, and classification bias may have influenced the observed associations. The association observed between fibromyalgia and glucocorticoid exposure should also be interpreted cautiously, as corticosteroid use in cross-sectional analyses may reflect symptom-driven treatment decisions or the misattribution of fibromyalgia-related symptoms to inflammatory disease activity rather than a causal role of corticosteroids in fibromyalgia development. although current prednisone use and dose were recorded and included in univariate regression analyses, glucocorticoid exposure may still have influence immune cell distribution, cytokine levels, and symptom burden, and residual confounding by prednisone cannot be excluded. Other prior biologic therapy could theoretically influence circulating immune profiles, however only three participants had previously received B-cell depletion therapy with rituximab approximately one year before enrollment, and no significant association with fibromyalgia status was observed. Nevertheless, a potential confounding effect cannot be completely excluded. Furthermore, information regarding the use of analgesic or neuromodulatory medications for chronic pain was not systematically recorded, and therefore their potential influence on pain perception could not be evaluated. Finally, given that the main associations reported in this study were derived from multivariate logistic regression analyses, the relatively small sample size and limited number of fibromyalgia cases represent an additional limitation. These conditions may contribute to model instability and wide confidence intervals for some variables, reflecting limited statistical power. Accordingly, the results should be interpreted cautiously and considered exploratory and hypothesis-generating, requiring confirmation in larger multicenter cohorts including more diverse IIM populations.

In conclusion, fibromyalgia represents a relevant and potentially underrecognized comorbidity in patients with idiopathic inflammatory myopathies, even during clinical remission or complete response. In our cohort, fibromyalgia was associated with a higher perceived disease burden and with distinct immunological and metabolic patterns, suggesting that nociplastic pain in IIM may involve specific biological pathways beyond residual inflammation. These findings highlight the importance of systematically evaluating nociplastic pain syndromes in clinical practice to avoid misattributing fibromyalgia-related symptoms to persistent inflammatory disease activity. Future studies integrating immunologic and metabolomic profiling in larger cohorts may help clarify the mechanisms underlying pain sensitization in IIM and improve patient stratification.

Acknowledgements: The authors acknowledge the technical support provided by the Red de Apoyo a la Investigación (RAI).

## Data Availability

The raw data supporting the conclusions of this article will be made available by the authors, without undue reservation.
