# Supplementary material for: Clinical, immunological and metabolomic risk factors associated with fibromyalgia in a cohort of patients with idiopathic inflammatory myopathies
Source: Front Immunol. 2026 Apr 24;17:1787182. doi: 10.3389/fimmu.2026.1787182 (PMC13153059; doi:10.3389/fimmu.2026.1787182)
Supplement: Supplementary file 1 [file SupplementaryFile1.pdf]

Supplementary material

Supplementary Figure S1. PLS-DA analysis comparing healthy donors (HD) and patients with idiopathic inflammatory myopathies and fibromyalgia (IIM + FM)

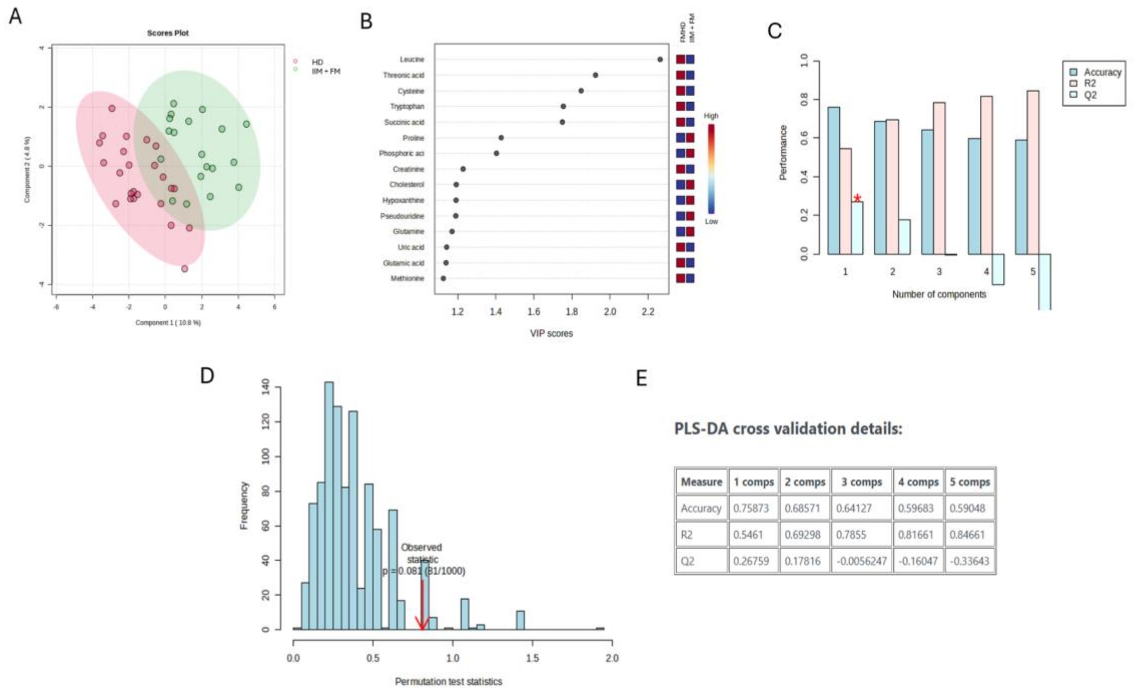

Measure	1 comps	2 comps	3 comps	4 comps	5 comps
Accuracy	0.75873	0.68571	0.64127	0.59683	0.59048
R2	0.5461	0.69298	0.7855	0.81661	0.84661
Q2	0.26759	0.17816	-0.0056247	-0.16047	-0.33643

A) Partial least squares discriminant analysis (PLS-DA) scores plot shows partial separation between healthy controls (pink) and IIM with FM patients (green) groups along Component 1 (10.8%) and Component 2 (4.8%).

B) Variable importance in projection (VIP) scores of top discriminatory metabolites between groups, highlighting leucine, threonic acid, cysteine, tryptophan, and succinic acid as most influential.

C) Bar plot of cross-validation metrics (Accuracy, R², Q²) across PLS-DA components, with best performance observed for two components.

D) Permutation test shows borderline statistical significance (p = 0.081), suggesting limited but non-random group separation.

E) PLS-DA cross-validation summary.

Supplementary Figure S2. PLS-DA analysis comparing patients with idiopathic inflammatory myopathies with FM (IIM + FM) and without FM (IIM).

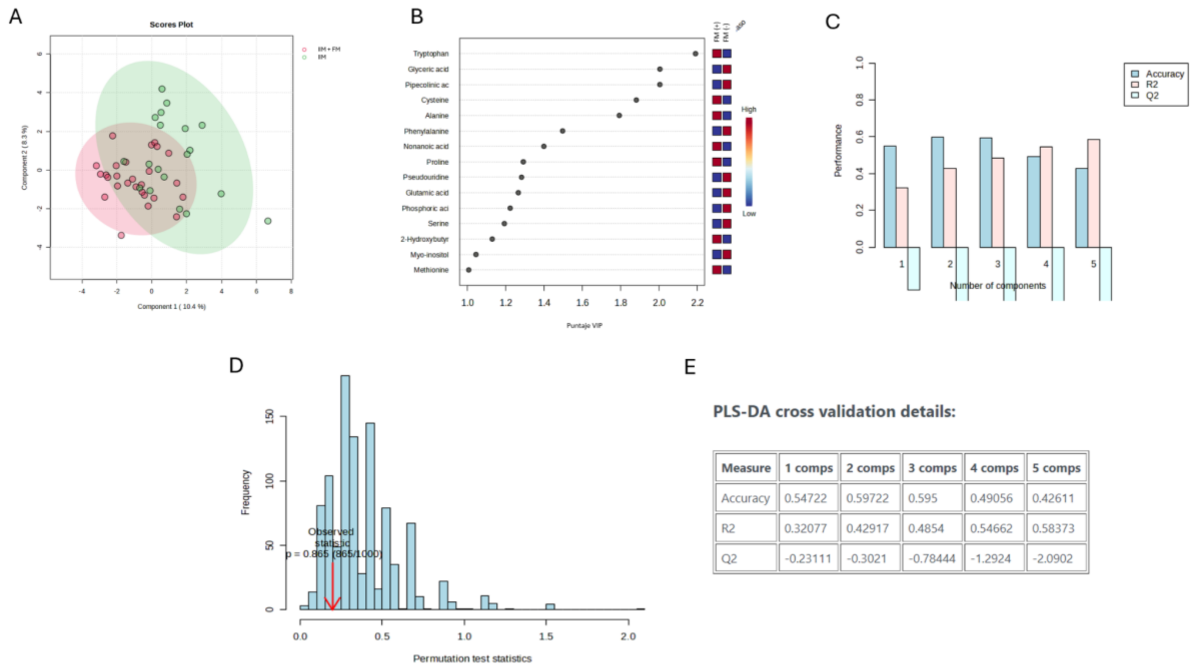

Measure	1 comps	2 comps	3 comps	4 comps	5 comps
Accuracy	0.54722	0.59722	0.595	0.49056	0.42611
R2	0.32077	0.42917	0.4854	0.54662	0.58373
Q2	-0.23111	-0.3021	-0.78444	-1.2924	-2.0902

A) PLS-DA scores plot shows overlap between IIM without FM (pink) and IIM with FM (green) groups, with minimal separation along Component 1 (10.4%) and Component 2 (8.3%).

B) VIP scores plot shows the top ranked metabolites, including tryptophan, oxopentanoic acid, and peptide-related intermediates; however, these did not yield clear group differentiation.

C) Cross-validation performance is poor, with low accuracy and negative Q^2 values across components.

D) Permutation test was not statistically significant ($p = 0.331$), indicating no robust metabolic separation between patients with IIM with or without FM.

E) Summary table of model metrics across 1–5 PLS components.

Supplementary Figure S3. Representative flow cytometry plots of immune subpopulations in IIM + FM and IIM without FM.

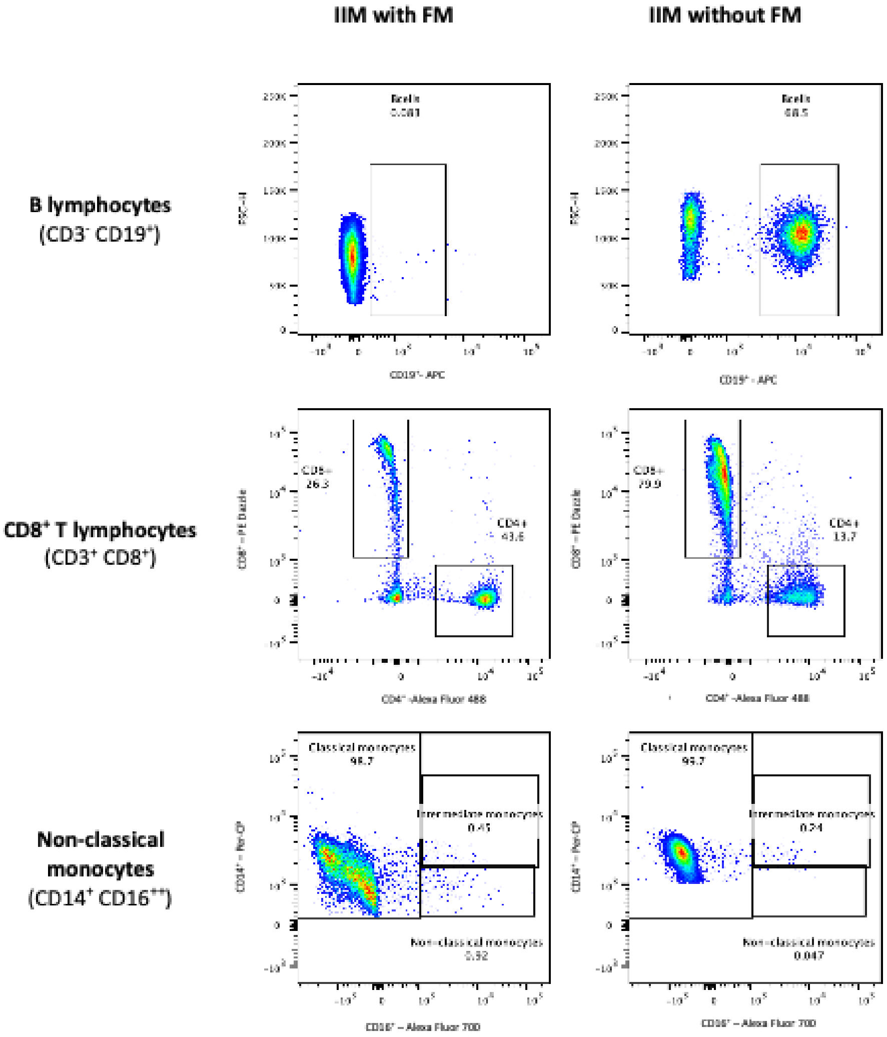

Representative gating strategy and dot plots from flow cytometry analysis showing B lymphocytes ($CD3^{-}CD19^{+}$), $CD8^{+}$ T lymphocytes ($CD3^{+}CD8^{+}$), and non-classical monocytes ($CD14^{+}CD16^{++}$) in patients with idiopathic inflammatory myopathies (IIM) with (left panels) and without (right panels) fibromyalgia (FM). The plots illustrate decreased abundance of these subsets in the FM+ group, consistent with quantitative findings reported.
